# Genome-wide analysis of auxin response factor gene family members in medicinal model plant *Salvia miltiorrhiza*

**DOI:** 10.1242/bio.017178

**Published:** 2016-05-26

**Authors:** Zhichao Xu, Aijia Ji, Jingyuan Song, Shilin Chen

**Affiliations:** 1 Institute of Medicinal Plant Development, Chinese Academy of Medical Science, Peking Union Medical College, Beijing 100193, China; 2 Key Laboratory of Bioactive Substances and Resources Utilization of Chinese Herbal Medicine, Ministry of Education, Beijing 100193, China; 3 Institute of Chinese Materia Medica, Chinese Academy of Chinese Medical Science, Beijing 100700, China

**Keywords:** Developmental processes, Auxin response factors, Auxin response elements, MicroRNA, *Salvia miltiorrhiza*

## Abstract

Auxin response factors (ARFs) can function as transcriptional activators or repressors to regulate the expression of auxin response genes by specifically binding to auxin response elements (AuxREs) during plant development. Based on a genome-wide strategy using the medicinal model plant *Salvia miltiorrhiza*, 25 *S. miltiorrhiza ARF* (*SmARF*) gene family members in four classes (class Ia, IIa, IIb and III) were comprehensively analyzed to identify characteristics including gene structures, conserved domains, phylogenetic relationships and expression patterns. In a hybrid analysis of the phylogenetic tree, microRNA targets, and expression patterns of SmARFs in different organs, root tissues, and methyl jasmonate or indole-3-acetic acid treatment conditions, we screened for candidate SmARFs involved in various developmental processes of *S. miltiorrhiza*. Based on this analysis, we predicted that SmARF25, SmARF7, SmARF16 and SmARF20 are involved in flower, leaf, stem and root development, respectively. With the further insight into the targets of *miR160* and *miR167*, specific *SmARF* genes in *S. miltiorrhiza* might encode products that participate in biological processes as described for *ARF* genes in *Arabidopsis*. Our results provide a foundation for understanding the molecular basis and regulatory mechanisms of SmARFs in *S. miltiorrhiza*.

## Abstract (precis)

**Summary:** Genome-wide analysis identified 25 ARF gene members (seven transcriptional activators and 18 repressors) in *S. miltiorrhiza*. The gene structures, functional domains, miRNA targets and expression patterns were analyzed in detail.

## INTRODUCTION

The phytohormone auxin, typified by indole-3-acetic acid (IAA), plays a crucial role in controlling the mechanisms by which plants grow and develop, including tropic responses, apical dominance, lateral root formation, vascular differentiation, flower and fruit development, and shoot elongation (Santner and Estelle, 2009). Auxin response factors (ARFs) are important transcription factors that can either activate or repress the transcriptional level of early/primary auxin response genes, such as *Aux/IAA*, *Small Auxin Up RNA* (*SAUR*) and *Gretchen Hagen 3* (*GH3*) gene family members, by binding to auxin response elements (AuxREs, TGTCTC) or some variation of these elements (TGTCCC or TGTCAC) in their promoters (Hagen and Guilfoyle, 2002; Liu et al*.*, 1994; Ulmasov et al*.*, 1997, 1995, 1999b). AtARF1, which binds to the sequence TGTCTC in AuxREs, was the first cloned auxin-related transcription factor and was identified in *Arabidopsis* using a yeast one-hybrid system (Ulmasov et al*.*, 1997). Recently, microarray experiments indicated that AtARF1 and AtARF5 monomers specificity prefer TGTCGG elements to the AuxRE TGTCTC (Boer et al*.*, 2014). The complete genomic sequence of *Arabidopsis* provides the opportunity to identify the sequence and evolution of all members of a given gene family (Arabidopsis Genome Initiative, 2000). Genome-wide analysis identified 22 full-length ARF genes and one partial-length gene (*AtARF23*) containing a stop codon in its DNA-binding domain (DBD) in *Arabidopsis thaliana* (Okushima et al*.*, 2005b; Remington et al*.*, 2004). Furthermore, biochemical and genetic approaches have established crucial functions of ARF genes in the growth and development of *Arabidopsis* (Guilfoyle and Hagen, 2007).

Taking advantage of the genome-wide identification of *A. thaliana* ARFs (AtARFs), many studies have found that the ARFs AtARF1 and AtARF2 function as transcriptional repressors related to the regulation of leaf senescence, floral organ abscission and cell growth (Ellis et al*.*, 2005; Li et al*.*, 2004; Okushima et al*.*, 2005a; Schruff et al*.*, 2006); AtARF3 and AtARF4 function in developing reproductive and vegetative tissues (Pekker et al*.*, 2005; Sessions et al*.*, 1997; Finet et al., 2010); AtARF5 functions in *Arabidopsis* leaf vascular and embryo patterning (Hamann et al*.*, 2002; Krogan et al*.*, 2012); AtARF6 and AtARF8 function in female and male reproduction (Nagpal et al*.*, 2005; Wu et al*.*, 2006); AtARF7 and AtARF19 act in seedlings, roots and developing embryos (Korasick et al*.*, 2014; Okushima et al*.*, 2005b; Wilmoth et al*.*, 2005); AtARF9 acts in suspensor cells to mediate hypophysis specification (Rademacher et al*.*, 2012); and AtARF10, AtARF16 and AtARF17 function in the negative regulation of seed germination and post-germination activities (Liu et al*.*, 2007, 2010). In *Arabidopsis*, certain *AtARF* expression patterns are controlled by miRNAs to regulate several developmental events. *AtARF6* and *AtARF8* are targets of *miR167* (Wu et al*.*, 2006), and *AtARF10*, *AtARF16* and *AtARF17* are targets of *miR160* (Liu et al*.*, 2007, 2010). In some cases, *ARF* gene expression is altered in response to exogenous auxin signals (Okushima et al*.*, 2005b; Wang et al*.*, 2007a).

A typical ARF contains three conserved domains: an N-terminal B3 DNA binding domain (DBD), a middle regional auxin response factor (MR), and a C-terminal PB1 protein-protein interaction domain (PB1). The DBD can recognize AuxREs or variation elements in the promoter of auxin-responsive genes (Wright and Nemhauser, 2015; Boer et al*.*, 2014), and the PB1 domains are also found in Aux/IAAs (Guilfoyle and Hagen, 2012). Structural and biochemical studies have determined that the PB1 domains of ARFs and Aux/IAAs from AtARF7 (Korasick et al*.*, 2014) and AtARF5 (Nanao et al*.*, 2014) are involved in protein-protein interactions by formatting higher order oligomerization or multimerization (Wright and Nemhauser, 2015). The MR, located between the DBD and the PB1 domain, confers functions such as transcriptional activation or repression depending on its amino acid composition (Mun et al*.*, 2012; Yu et al*.*, 2014). Previous studies have shown that glutamine (Q)-rich MRs function as activation regions but that serine (S)-rich, serine and proline (SP)-rich, and serine and glycine (SG)-rich MRs function as repression regions in ARFs from *A. thaliana* (Tiwari et al*.*, 2003; Ulmasov et al*.*, 1999a).

Given the complete genomic sequences of many important species, there has been significant progress in the analysis and identification of the functions of ARFs. Genome-wide analysis has identified many ARFs in many other important plants, such as 25 *Oryza sativa* ARF (OsARF) loci (Wang et al*.*, 2007a), 22 *Solanum lycopersicum* ARFs (SlARFs) (Zouine et al*.*, 2014), 31 *Brassica rapa* (BrARFs) (Mun et al*.*, 2012), 19 *Vitis vinifera* ARFs (VvARFs) (Wan et al*.*, 2014), 47 *Musa acuminata* ARFs (MaARFs) (Hu et al*.*, 2015), 17 *Eucalyptus grandis* (EgrARFs) (Yu et al*.*, 2014), 24 *Medicago truncatula* ARFs (MtARFs) (Shen et al*.*, 2015), 39 *Populus trichocarpa* ARFs (PtARFs) (Kalluri et al*.*, 2007), 19 *Citrus sinensis* ARFs (CiARFs) (Li et al*.*, 2015b), 11 *Carica papaya* ARFs (CpARFs) (Liu et al*.*, 2015), and 35 *Gossypium raimondii* ARFs (GrARFs) (Sun et al*.*, 2015). However, the ARF transcription factor family members have not been determined in *Salvia miltiorrhiza*, one of the most commonly used herbs in traditional Chinese medicine (TCM). *S. miltiorrhiza*, also referred to as danshen, belongs to the *Salvia* genus of the Lamiaceae family, and its dried root and rhizome are highly valued (Cheng, 2006). Danshen is well known for its use alone or in combination with other herbs in the treatment of cardiovascular diseases, as well as for its anti-inflammatory, immunomodulatory and anti-oxidative activities; the primary bioactive compounds in danshen are lipophilic diterpenoids and hydrophilic phenolic acids (Wang et al., 2007b; Dong et al*.*, 2011). *S. miltiorrhiza* is also considered a good medicinal model plant in TCM research for studying the biosynthesis and regulation of active compounds (Ma et al*.*, 2012; Xu et al*.*, 2015). Due to the establishment of the *S. miltiorrhiza* genome sequence (Xu et al*.*, 2016a) it has become feasible use *in silico* analysis to isolate its functional gene families such as diterpene; phenolic acid biosynthetic genes; and bHLH, AP2/ERF, WRKY, MYB and SPL transcription factors (Ji et al., 2015; Li et al*.*, 2015a; Li and Lu, 2014; Ma et al*.*, 2012; Wang et al*.*, 2015; Zhang et al*.*, 2014, 2015; Xu et al., 2016b). As ARF gene members are key factors in plant growth and development, identifying these genes in *S. miltiorrhiza* aid in the understanding of developmental processes and cellular responses to auxin in danshen.

Here, we isolated 25 *S. miltiorrhiza* ARF (*SmARF*) genes using a genome-wide approach. Following complete genome sequencing the sequence homology of these *SmARFs* and their gene expression patterns in different organs, root tissues, and methyl jasmonate (MeJA) or IAA treatment conditions, gene structures, and the phylogenetic relationships between *SmARFs* and *AtARFs* were analyzed in detail. This study provided molecular information regarding the *SmARF* gene family and the results will aid in selecting candidate genes related to cell growth and tissue development in *S. miltiorrhiza*, paving the way for further functional characterization of these *SmARF* genes.

## RESULTS

### Identification and phylogenetic analysis of danshen ARFs

After a BLASTP search and protein domain analysis, 25 non-redundant *ARF* genes were identified from the genome sequences of *S. miltiorrhiza*. These *SmARFs*, located in the different scaffolds, were named *SmARF1*-*SmARF25* according to the order of their annotated gene IDs, listed in Table 1. The number of *ARF* genes in *S. miltiorrhiza* is similar to the number in *A. thaliana* (23), *O. sativa* (25) and *M. acuminata* (24). The predicted proteins encoded by *SmARF* genes varied from 345 amino acids (SmARF12) to 1105 amino acids (SmARF22), with corresponding molecular weights from 37.78 kDa to 122.17 kDa, and the theoretical isoelectric points ranged from 5.29 (SmARF5) to 9.28 (SmARF12). Pair-wise analysis of SmARF protein homology indicated that the overall homology broadly ranged from 22% (between SmARF6 and SmARF16) to 89% (between SmARF5 and SmARF7). The *SmARF9* and *SmARF10* genes are located in the same scaffold1069, and the other *SmARFs* are distributed in different scaffolds. Most of the SmARFs were predicted to localize to the nucleus, however SmARF1 and SmARF12 were predicted to localize to chloroplasts.

**Table 1.**
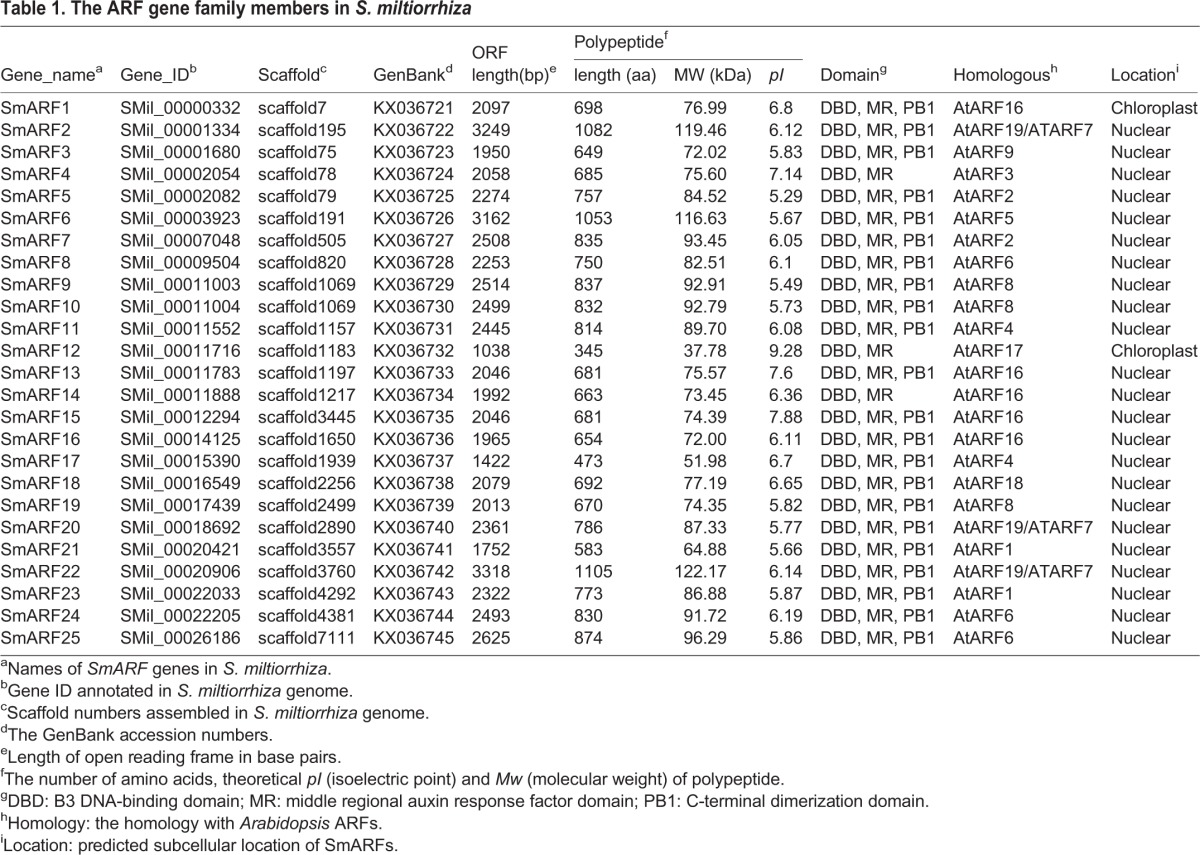
**The ARF gene family members in *S. miltiorrhiza***

**Fig. 1.**
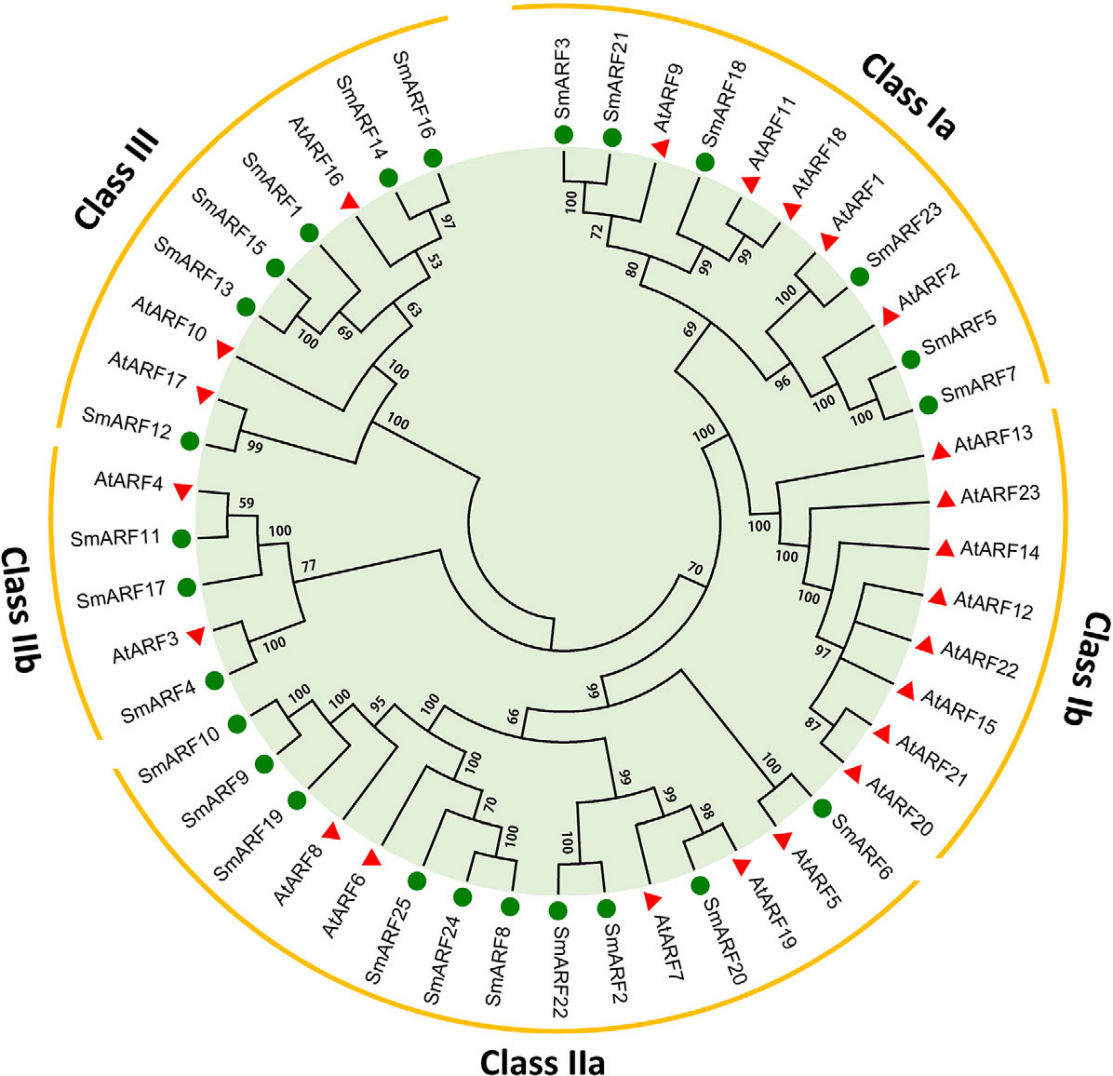
**Analysis of the phylogenetic relationships of *ARF* gene members in *S. miltiorrhiza* and *Arabidopsis*.** A total of 25 SmARF proteins from *S. miltiorrhiza* and 23 ARF proteins from *Arabidopsis* were used to construct a neighbor-joining tree. Bootstrap values are presented for all branches. The 25 SmARFs and 23 AtARFs were clustered into five classes (Ia, Ib, IIa, IIb and III). Green circles denote the ARF proteins from *S. miltiorrhiza*, and red triangles denote the ARF proteins from *Arabidopsis*.

To characterize the evolutionary relationship between danshen ARF proteins and *Arabidopsis* ARFs, a neighbor-joining tree was constructed using the full-length amino acid sequences (Fig. 1). The results indicated that 25 SmARFs were classed together with 23 AtARFs into four clusters (classes Ia, IIa, IIb and III) according to well-supported bootstrap data. In *S. miltiorrhiza*, SmARF3, 5, 7, 18, 21 and 23 belong to class Ia; SmARF2, 6, 8, 9, 10, 19, 20, 22, 24 and 25 belong to the largest class IIa; SmARF4, 11 and 17 belong to class IIb; and SmARF1, 12, 13, 14, 15 and 16 belong to class III. In *A. thaliana*, there is another class Ib that includes AtARF12-15 and 20-23. Notably, no *S. miltiorrhiza* ARF proteins were clustered into class Ib from the phylogenetic tree, and this observation implies a diverging trend in the evolution of ARF genes across different plants.

To investigate the biological processes of SmARFs, gene ontology (GO) mapping and annotation were performed using Blast2GO. The functional categorization of SmARFs as annotated by GO analysis, including their biological processes, molecular functions, and cellular components, is presented in Table S1. Regarding biological processes, eight categories met the criterion of NodeScore >2.0: cellular process (25 genes), metabolic process (25 genes), response to stimulus (25 genes), single-organism process (14 genes), biological regulation (13 genes), signaling (11 genes), developmental process (five genes), and multicellular organismal process (five genes). For developmental process, SmARF6 was related to embryo development; five SmARFs were classified into post-embryonic developmental process (SmARF1, 4, 6, 11, 25); and SmARF1, 4, 6 and 25 were predicted to participate in flower development. Notably, the biological process related to secondary metabolism was not identified. Based on the molecular function analysis, all the SmARFs were classified into DNA binding; 21 SmARFs were grouped into protein binding; and three SmARFs were categorized into sequence-specific DNA binding transcription factor activity (SmARF1, 11 and 25). According to the cellular component analysis, all SmARFs except for SmARF1 and SmARF12 were localized to the nucleus, in accordance with the subcellular localization predictions.

### Gene structures and conserved domains of danshen ARFs

**Fig. 2.**
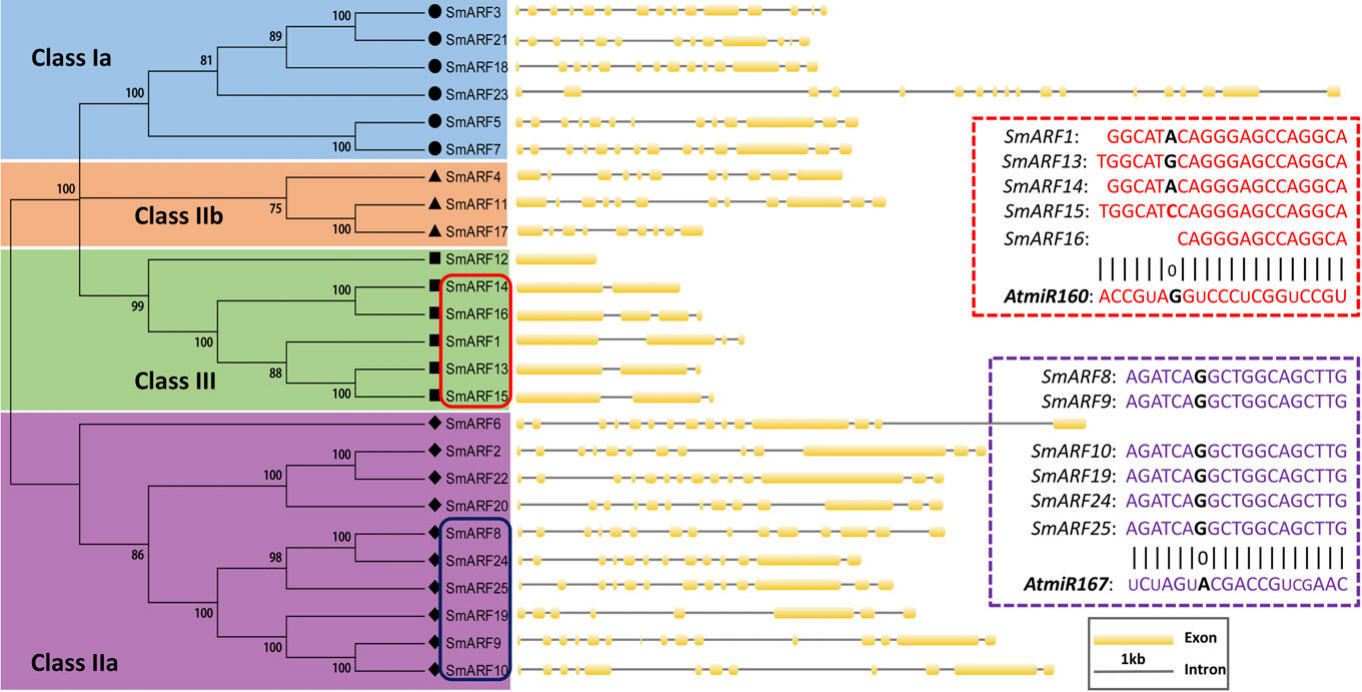
**Gene structure analysis of *SmARF* genes and prediction of their miRNA target sites according to their phylogenetic relationships.** The yellow boxes represent exons; the gray lines represent introns. The red box denotes the targets of *At-miR160* in *SmARF* genes; the purple box denotes the targets of *At-miR167* in *SmARF* genes.

To better understand the gene structure of SmARFs, the exon-intron features among SmARFs were aligned via phylogenetic analysis (Fig. 2). The phylogenetic analysis revealed four clusters in accordance with the group data presented in Fig. 1. Gene structure analysis of all of the *SmARF* genes revealed that the number of exons ranges from 1 to 18, however, *SmARF12* is intronless. The genes in the four groups have an average exon number ranging from three (class III) to 15 (class Ia). The results showed that the exon number of class I-II was significantly greater than that of class III; these findings were identical to the structure of *AtARF* genes.

Examination of the protein homology of SmARFs to *Arabidopsis* ARFs showed that 10 AtARFs have no corresponding *S. miltiorrhiza* orthologs (AtARF10-15 and AtARF20-23). Sequence analysis and Pfam protein domain analysis showed that 92% of the identified SmARFs (23 of the 25 predicted proteins) possess the typical ARF structure, containing a highly conserved DBD, MR and PB1. In contrast to the typical ARFs, SmARF4, 12 and 14 do not contain a PB1 domain. ARFs function as transcriptional activators or repressors depending on the amino acid composition of the MR. The Q-rich MRs of seven SmARFs (SmARF2, 10, 19, 20, 22, 24 and 25) (Table S2, Fig. S1) indicate that they likely act as transcriptional activators. The SmARFs containing Q-rich MR domains belong to class IIa according to phylogenetic analysis. The other 18 SmARFs may function as transcriptional repressors based on their S-rich, SP-rich, or SG-rich MRs.

A total of 15 conserved motifs in SmARFs were characterized (motifs 1-15) using MEME software to explore their structure and functional diversity (Figs S2 and S3). SmARF2, 3, 7, 8, 9, 18, 22 and 24 in classes Ia and IIa contain the greatest number of distinct motifs (12), and SmARF12 in class III contains the fewest distinct motifs (six types). Additionally, the average motif number per SmARF varies across classes, ranging from nine (class IIb and III) to 11 (class Ia and IIa). This evidence indicated that the ARF proteins are highly conserved. Motifs 1, 3 and 12 were annotated as B3-DBDs; motifs 4, 6, 10 and 11 were annotated as auxin response superfamily MRs; and motifs 7 and 8 were annotated as the OPCA-like motif and conserved lysine motif of PB1 domain, which function in Aux/IAA-ARF multimerization. In accordance with the results of conserved domain analysis, all SmARF protein structures harbor DBD motifs (1, 3 and 12) and MR motifs (4, 6, 10 and 11); however SmARF4 and SmARF12 do not contain a PB1 (neither motif 7 nor motif 8).

### Prediction of miRNA targets among *SmARFs* and analysis of the AuxREs in *SmARF* gene promoters

Using the BLASTN algorithm to identify targets of *miRNA160* and *miRNA167* within *SmARF* gene sequences, target sites of *At-miRNA160* (UGCCUGGCUCCCUGGAUGCCA) were predicted within the 1300-1319 bp region of *SmARF1*, the 1359-1379 bp region of *SmARF13*, the 1348-1367 bp region of *SmARF14*, the 1332-1352 bp region of *SmARF15* and the 1363-1376 bp region of *SmARF16*. Additionally, target sites of miRNA167 (UCAAGCUGCCUGCAUGAUCUA) were predicted within the 1975-1993 bp region of *SmARF8*, the 2302-2320 bp region of *SmARF9*, the 2287-2305 bp region of *SmARF10*, the 1777-1795 bp region of *SmARF19*, the 2419-2437 bp region of *SmARF24* and the 2350-2368 bp region of *SmARF25* (Fig. 2). The results of this analysis suggested that miR160-/167-mediated post-transcriptional regulation of ARFs is conserved between *S. miltiorrhiza* and *Arabidopsis*.

We surveyed 20 *AUX/IAA* and 10 *GH3* primary/early auxin response gene members in *S. miltiorrhiza* based on a genome-wide strategy. The promoters (−1000 to −1 bp) of these two auxin response gene families were selected to screen for AuxREs. As expected, 19 of 20 *AUX/IAA* and 9 of 10 *GH3* gene promoters contain one or more AuxREs (Table S3). These results indicated that these auxin response genes could be regulated by SmARFs in *S. miltiorrhiza*.

### Expression patterns of *SmARF* genes in different plant organs or tissues

**Fig. 3.**
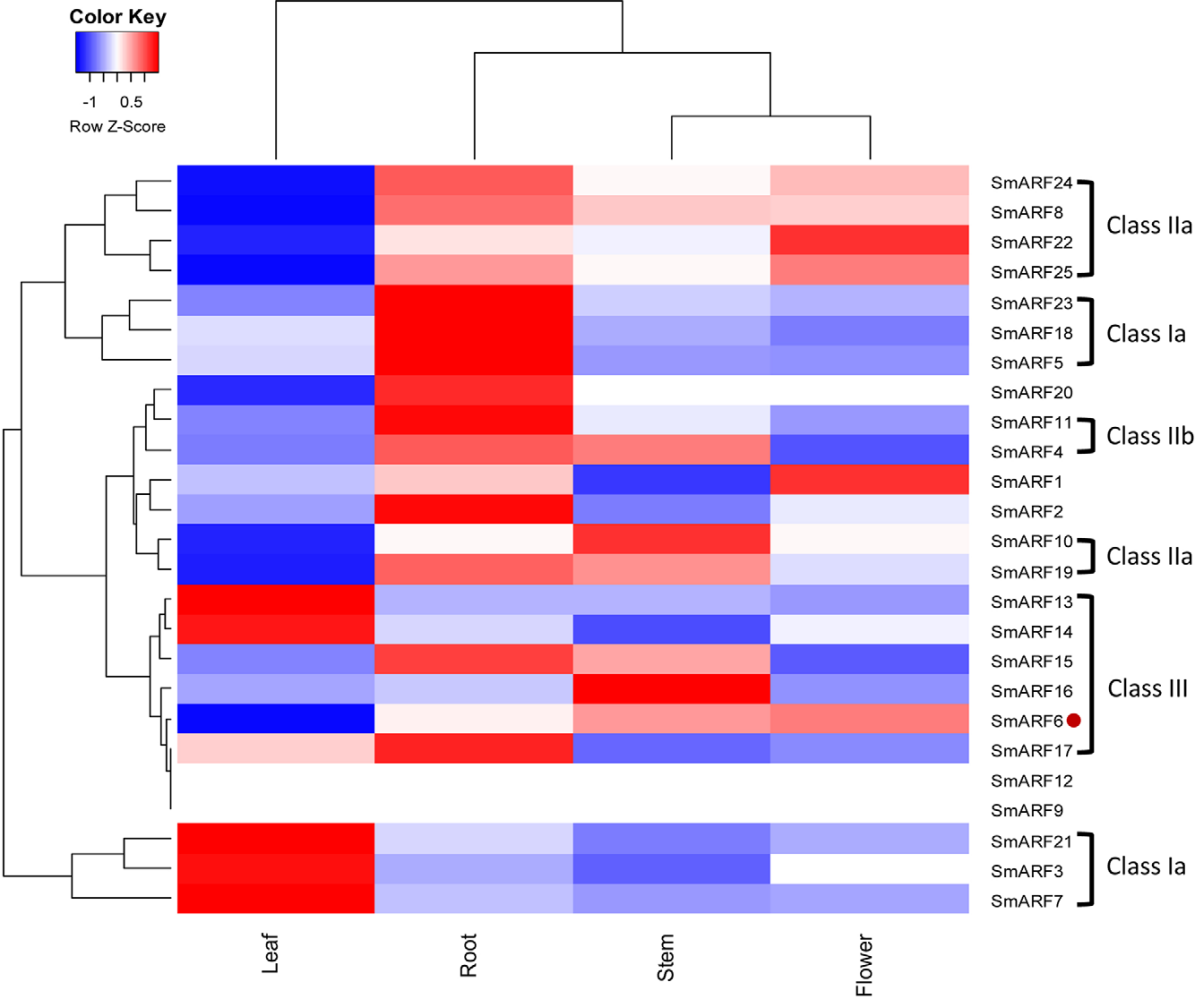
**A heat map showing *SmARF* gene expression patterns in different organs.** The red color represents upregulation of expression, the white color represents an unchanged expression level, and the blue color represents downregulation of expression. Red dot (SmARF6) is not belong to the Class III.

To better probe the physiological function of SmARFs, the tissue-specific expression of 25 *SmARF* genes in different danshen organs (leaf, root, stem and flower) was determined by analyzing the RNA-seq data (Fig. 3; Table S4). Most *SmARF* genes, but not *SmARF9*, *12* or *17* presented ubiquitous expression and high variability in all studied organs, and this result implies that these SmARFs might function in danshen growth and development. There were significant differences in SmARF expression between organs. *SmARF3*, *7* and *21*, all of which belong to class Ia based on phylogenetic analysis, showed higher expression in danshen leaf than in other organs. *SmARF4*, *5*, *11*, *18*, *20* and *23* were expressed more strongly in danshen root than in other organs, however, only *SmARF16* showed stem-specific expression in *S. miltiorrhiza*. When comparing phylogenetic tree analysis with the expression cluster analysis, *SmARF8*, *10*, *19*, *22*, *24* and *25*, which belong to class IIa, showed significantly lower expression in danshen leaf than in other organs. Most of the *SmARF* genes from class III (*SmARF13-17*) also clustered in one expression branch. These results indicated that ARF genes from the same class might perform a similar physiological function in plants.

Previous evidence revealed that the periderm of danshen root is the primary site of biosynthesis and accumulation of tanshinones. The expression pattern of *SmARF* genes in different root tissues (periderm, phloem and xylem) was also examined using RNA-seq data (Fig. S4, Table S4). *SmARF9*, *12* and *17* displayed no expression in danshen root tissues. *SmARF13* showed the greatest expression in periderm, more than four and 14 times greater than that in phloem and xylem, respectively. Furthermore, *SmARF20* exhibited stronger expression in phloem and xylem than in periderm.

### Expression patterns of *SmARF* genes upon auxin or MeJA treatment

**Fig. 4.**
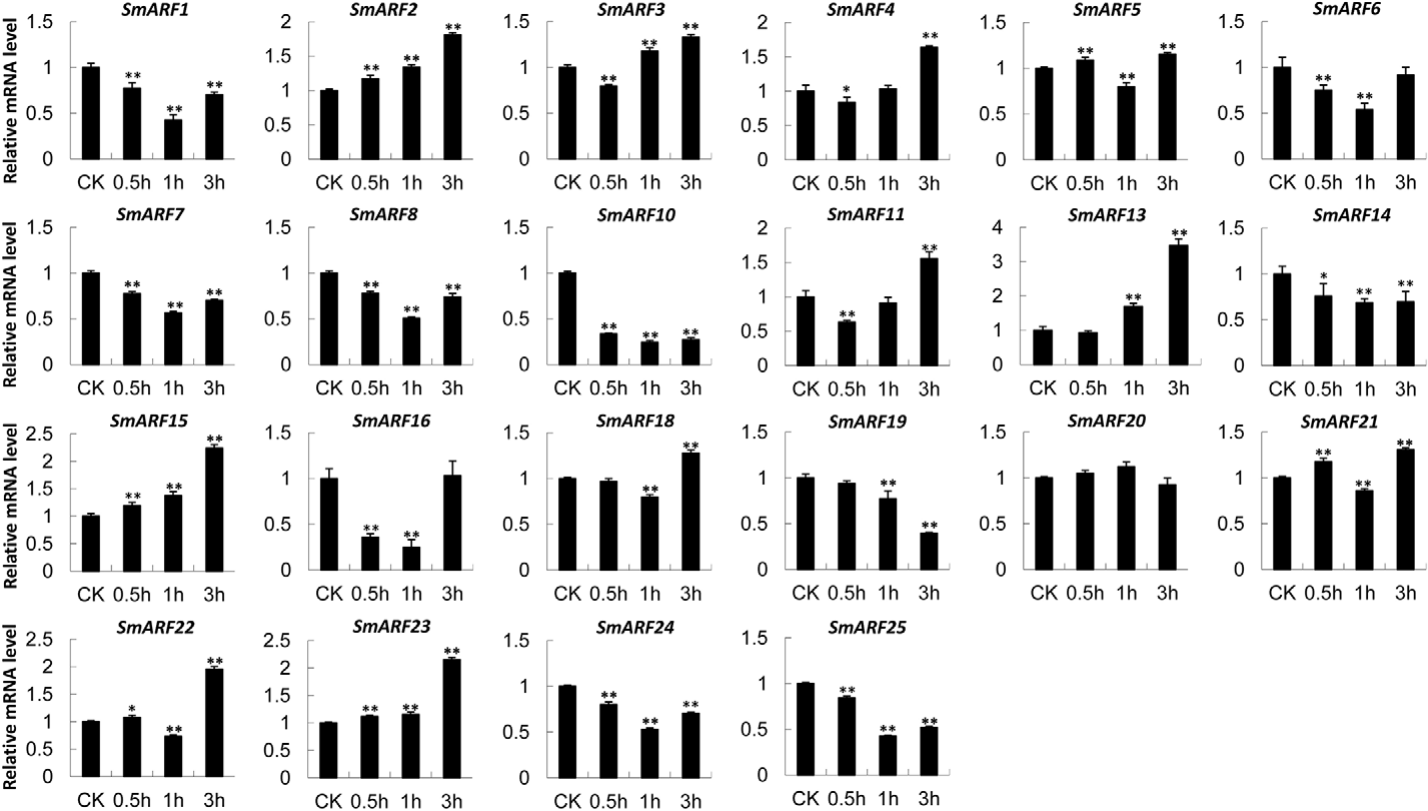
**The expression of *SmARF* genes in response to treatment with 20 μM IAA solution for 0.5, 1, or 3 h.** CK, the untreated leaves of *S. miltiorrhiza*. Error bars represented variability of qRT-PCR results from three replicates. No expression of *SmARF9*, *12* and *17* was detected. *P<0.05; **P<0.01.

Auxin is a central regulator of plant growth and development. To investigate the response of *SmARF* genes to exogenous IAA stimulation, we analyzed the variation in SmARF gene expression at 0, 0.5, 1 and 3 h after 20 μM IAA treatment using qRT-PCR (Fig. 4). As expected, most SmARF genes were significantly auxin-sensitive. The overall expression patterns of SmARFs varied, with 11 SmARF mRNAs (*SmARF2*, *3*, *4*, *5*, *11*, *13*, *15*, *18*, *21*, *22* and *23*) showing up-regulation and eight SmARF mRNAs (*SmARF1*, *7*, *8*, *10*, *14*, *19*, *24* and *25*) showing down-regulation at 3 h of IAA treatment (*P*<0.01 for all). One SmARF gene (*SmARF20*) did not display significant changes in expression (*P*>0.05) regardless of the treatment duration. The unmentioned *SmARF6* and *SmARF16* displayed significantly down-regulated expression at 0.5 and 1 h, and at 3 h, the expression of these genes returned to the same level as that for mock IAA treatment. The most strongly up-regulated *SmARF* genes, *SmARF13*, *15* and *23*, were markedly induced after IAA treatment [greater than twofold increase, log (expression level) >1]. Similarly, the expression of five *SmARF* genes (*SmARF1*, *10*, *16*, *19*, *25*) showed marked down-regulation [greater than twofold decrease, log (expression level) >1]. For IAA treatment, 13 *SmARF* genes (*SmARF1*, *3*, *5*, *6*, *7*, *8*, *11*, *16*, *18*, *21*, *22*, *24* and *25*) displayed significant up- or downregulation over the three examined time points. For example, the expression level of SmARF1 was decreased by greater than twofold at 1 h but was significantly increased at 3 h compared with the control levels.

In *Nicotiana benthamiana*, transient silence of *NbARF1* and MeJA treatment resulted in significant enrichment of leaf nicotine (Todd et al., 2010). In addition, MeJA treatment significantly alters the biosynthesis of active compounds (tanshinones or phenolic acids) in *S. miltiorrhiza*, hence the expression variation of *SmARF* genes after MeJA treatment was studied using RNA-seq data (Table S4). The results showed that *SmARF24* and *25* displayed significant up-regulation and that *SmARF1* exhibited downregulation after MeJA treatment. The expression of other SmARF genes showed no evident changes following MeJA treatment. This finding suggests that SmARFs might perform a small role in post-developmental processes.

## DISCUSSION

The cultivation of medicinal plants has faced intense pressure due to social and environmental concerns. Studying the molecular mechanisms of medicinal plant growth processes would help resolve potential questions related to cultivation of these plants. Genome-wide characterization and analysis of SmARFs could improve the understanding of their regulatory roles in danshen growth and development. In this study, 25 *ARF* gene members in *S. miltiorrhiza* were identified, and this number was similar to that for other model plants, such as *A. thaliana* (23) and *O. sativa* (25). Protein domain analysis provided useful information for predicting the biological functions of SmARFs, which primarily depend on their characteristic DBD, MR and PB1. ARFs rely on the DBD to specifically bind to AuxREs in the promoters of auxin-responsive genes. Their PB1 is involved in homomeric and heteromeric interactions with ARFs and Aux/IAA proteins. The percentage of PB1-truncated SmARFs (8%) was much lower than that of the ARF members identified in other plants, such as *Arabidopsis* (17%), rice (24%) and *M. truncatula* (54%). ARFs can function as transcriptional activators or repressors according to the amino acid composition of the MR. The activator/repressor ratio of SmARFs was 0.39 (7/18) and this value was also much lower than the ratios for *Arabidopsis* (0.59) and rice (0.56). Phylogenetic analysis and divergence time estimation based on 1824 single-copy true orthologous genes indicated that *S. miltiorrhiza* was distantly related to *Arabidopsis*, with an estimated divergence time of approximately 139 million years ago (Xu et al., 2016a).We also constructed a phylogenetic tree to analyze the relationship of ARF family members between *S. miltiorrhiza* and *Arabidopsis* (Fig. 1). Phylogenetic tree analysis revealed five sister gene pairs with high bootstrap values (≥98%) between *S. miltiorrhiza* and *Arabidopsis*; this evidence supports the high homology of ARFs between species. Although there are similar numbers of ARFs between the *S. miltiorrhiza* and *A. thaliana* genomes, the absence of class Ib ARFs from *S. miltiorrhiza* reflects genomic expansion and rearrangements resulting from extensive duplication and deletion over a long period of evolutionary history according to the phylogenetic tree analysis. The detection of close relationships based on comparative analysis may help in the selection of candidate ARFs with specific biological functions in *S. miltiorrhiza*. According to the motif analysis, the motifs from different classes in SmARFs present high conservation (Fig. S2). Motif 8 and motif 7, located in the PB1 domain of the C-terminal in most SmARFs, include conserved residues (lysine motif and OPCA-like motif) of the positive and negative face found only in the ARF family, thus indicating the evolutionary conservation of ARF function (Korasick et al*.*, 2014; Nanao et al*.*, 2014).

Much evidence demonstrates that *miRNAs* play dominant roles in post-transcriptional gene regulation by binding to their complementary mRNA targets, especially to target transcription factors, in plants (Jones-Rhoades et al*.*, 2006; Li and Zhang, 2016). In *Arabidopsis*, *miR167* controls the expression patterns of *AtARF6* and *AtARF8* to regulate female and male reproduction or to promote jasmonic acid production and flower maturation (Nagpal et al*.*, 2005; Wu et al*.*, 2006). Phylogenetic tree analysis showed that SmARF8, 9, 10, 19, 24 and 25 were closely related to AtARF6 and AtARF8, both of which are in class IIa; all of these six SmARFs contain a target site of miR167 (Fig. 2). GO analysis also categorized SmARF25 into flower development; thus in *S. miltiorrhiza* we predicted that the expression of *SmARF8, 10*, *19*, *24* and *25* might be inhibited by *miR167* to regulate certain developmental processes as described for *AtARF6* and *AtARF8*. Among them, *SmARF25* was identified as the best candidate regulator of flower development. In addition, SmARF10, 19, 24 and 25 might function as transcriptional activators due to their Q-rich MRs. Additionally, miR160 was found to bind to AtARFs (AtARF10, AtARF16 and AtARF17) to negatively regulate seed germination and post-germination activities (Liu et al*.*, 2007, 2010). These AtARFs were closely related to SmARF1, 12, 13, 14, 15 and 16 in class III, and these ARFs might function as transcriptional repressors due to the amino acid compositions of their MRs. Aside from *SmARF12*, other class III *SmARF* genes were identified to contain *miR160* target sites; this finding implies that these SmARFs perform functions that are similar to the functions of AtARF10, 16 and 17.

Comprehensive analysis of *SmARF* gene expression patterns and the evolution of their sequences helped us screen for candidate *SmARF* genes with potentially distinct functions. Most *SmARF* genes displayed ubiquitous but highly variable expression in all studied organs, and this expression pattern suggests their functional divergence. In *Arabidopsis*, AtARF2 regulates leaf senescence and floral organ abscission independently of the ethylene and cytokinin response pathways (Ellis et al*.*, 2005). In *S. miltiorrhiza* the expression levels of *SmARF3*, *7* and *21* were significantly higher in leaves than in other studied organs, and SmARF5 and SmARF7 were closely related to AtARF2 in class Ia; these findings indicate that SmARF7 might play a crucial role in leaf development. In *Arabidopsis* AtARF7 and AtARF19 promote leaf expansion and auxin-induced lateral root formation (Wilmoth et al*.*, 2005). In *S. miltiorrhiza*, SmARF20 was grouped with AtARF7 and 19 in class IIa, and the expression of SmARF20 was much higher in danshen root than in other organs. These observations suggest that SmARF20 likely regulates auxin-induced lateral root formation. The differential expression of *SmARF20* between periderm, phloem, and xylem further support its role in root development. Notably, SmARF8, 10, 19, 22, 24 and 25 also belong to class IIa with AtARF7 and 19. The expression patterns of these SmARFs were much lower in danshen leaf, reflecting that they might be involved in leaf expansion. SmARF16, a stem-specifically expressed transcription factor, likely participates in stem development.

Recently, synthetic biology, particularly the biosynthesis of natural products, has advanced by leaps and bounds. The tanshinone and phenolic acid biosynthetic pathways, which have gradually been elucidated, have attracted increasing attention (Cui et al., 2015; Guo et al., 2013; Ma et al., 2012; Xu et al., 2015); however the molecular mechanism of danshen development has been an unpopular subject despite the importance of this medicinal plant. In this study the basic functional characteristics of SmARFs, such as the presence of the conserved DBD, MR and PB1 in 88% (22/25) of the SmARFs; the significant variation in the expression of 95% (21/22) of the examined SmARFs after 0.5 h, 1 h and 3 h of IAA treatment; and the presence one or more AuxREs in 85% (17/20) of the *AUX/IAA* gene promoters and 90% (9/10) of the *GH3* gene promoters in SmARFs indicated their regulatory roles in danshen growth and development. Further biochemical and genetic studies of candidate ARFs in *S. miltiorrhiza* will lead to the production of a working model for the cultivation and selective breeding of fine varieties of medicinal plants.

In summary, 25 ARF gene members (seven transcriptional activators and 18 repressors) in *S. miltiorrhiza* were identified, and a comprehensive account of this gene family has been performed. SmARFs were grouped into four classes with AtARFs in *Arabidopsis*, and the gene structures, functional domains, and miRNA targets of SmARFs were analyzed in detail. Expression patterns were used to predict candidate SmARFs involved in the regulation of various developmental processes. The results of this study will provide a basic foundation for the verification of the functions and evolution of *SmARF* gene family members in this model medicinal plant.

## MATERIALS AND METHODS

### Genome-wide survey of *ARF* genes in *S. miltiorrhiza*

The *Arabidopsis* ARF protein sequences (AtARF1 to AtARF23) were downloaded from the NCBI database (http://www.ncbi.nlm.nih.gov/protein/). BLASTP searches were used to identify the corresponding *ARF* gene members in *S. miltiorrhiza* using a cut-off e-value of 1.0E^−10^. The hidden Markov model (HMM) profiles of ARF gene family members including B3-DBD (Pfam02362), AUX_RESP (MR, Pfam06507), and AUX/IAA family (PB1, Pfam02309) members were applied to identify ARF genes based on the *S. miltiorrhiza* genome. The domains of all obtained ARFs were analyzed using BLAST from the Conserved Domain Database (http://www.ncbi.nlm.nih.gov/cdd). The auxin response genes, AUX/IAA gene family (Pfam02309) members and GH3 gene family (Pfam03321) members were also selected using the same approach. The Compute pI/Mw tool on the ExPASy server (http://web.expasy.org/compute_pi/) was employed to predict the theoretical isoelectric point (pI) and the molecular weight (Mw) of each SmARF protein.

### Gene structure, conserved motif and subcellular localization analyses

The Gene Structure Display Server (GSDS 2.0; http://gsds.cbi.pku.edu.cn/index.php) was used to analyze the gene structure of *SmARFs* with the input of coding sequences (CDSs) and corresponding genomic sequences. Conserved motifs in *SmARF* transcription factors were identified using MEME (Suite version 4.9.1; http://meme-suite.org/tools/meme) according to the following criteria: maximum number of 15 motifs and an optimum width of 8-50 amino acids. subCELlular LOcalization predictor (CELLO v.2.5; http://cello.life.nctu.edu.tw/) was used to predict the subcellular localization of SmARF proteins.

### Phylogenetic tree construction and miRNA target site analysis

All SmARF and AtARF protein sequences were pooled into MEGA6 (http://www.megasoftware.net/) to perform multiple sequence alignments. Then neighbor-joining trees were constructed using the bootstrap method with 1000 replications and pairwise deletion of gaps/missing data. The miRNA target sites of *AtmiR160* and *AtmiR167* in the *SmARFs* were searched using the PMRD database (http://bioinformatics.cau.edu.cn/PMRD/).

### Plant resources

*S. miltiorrhiza* (line 99-3) was cultivated at the Institute of Medicinal Plant Development (IMPLAD), Chinese Academy of Medical Sciences (CAMS), in an open experimental field. Three-year-old roots, stems, and flowers were collected. The roots were peeled into three parts (periderm, phloem and xylem) (Xu et al*.*, 2015). Leaves with or without MeJA treatment (12 h, 200 μM; Sigma-Aldrich, MO, USA) were collected from tissue culture plantlets of *S. miltiorrhiza* at 25°C under a long day of 16-h light/8-h dark (Zhang et al*.*, 2015). For auxin treatment, seedlings from tissue culture plantlets were incubated for 0.5 h, 1 h, or 3 h in 20 μM IAA solution. All of the collected tissues originated from an asexual line of *S. miltiorrhiza* 99-3.

### Sequencing data and bioinformatic analysis

The draft genome of *S. miltiorrhiza* was assembled and annotated in our lab [Xu et al., 2016a; Sequence Read Archive (SRA) accession number SRP051524, http://www.ncbi.nlm.nih.gov/sra]. The RNA-seq reads from different organs (root, stem and flower) were generated using Illumina HiSeq 2000 platforms (Illumina, USA; SRA accession number SRP028388). The RNA-seq reads from different root tissues (periderm, phloem and xylem) using Illumina HiSeq 2500 platforms (Illumina, USA) have been reported in our recent study (Xu et al*.*, 2015; SRA accession number SRR1640458). The Illumina reads from leaves with or without 12 h MeJA treatment were obtained from a previous study (Luo et al*.*, 2014; SRA accession number SRP051564). Differential *SmARF* gene expression in various root tissues, organs and treatment conditions was analyzed using Tophat 2.0.12 and Cufflinks 2.2.1 (Trapnell et al*.*, 2012) by mapping Illumina-derived short reads to the *S. miltiorrhiza* genomic sequence. A heat map was constructed using R statistical software (Le Meur and Gentleman, 2012). GO mapping and annotation were performed using Blast2Go with a cut-off e-value of 1.0E^−10^.

### Gene expression analysis by qRT-PCR

Four RNA samples of seedlings from tissue culture plantlets that were treated with IAA (mock, 0.5 h, 1 h, or 3 h) were isolated. Total RNA was isolated from three biological replicates for each sample using the RNeasy Plus Mini kit (Qiagen, Germany). Reverse transcription was performed using PrimeScript™ Reverse Transcriptase (TaKaRa, Japan). The qRT-PCR primers were designed using Primer Premier 6 (Table S5), and their specificity was verified by PCR. qRT-PCR analysis was conducted in triplicate using SYBR^®^ Premix Ex Taq™ II (TaKaRa, Japan), with *SmActin* as a reference gene, with a LightCycler 480 real-time PCR system (Roche, Switzerland). Ct values were calculated to analyze the relative expression levels using the 2^−ΔΔCt^ method (Livak and Schmittgen, 2001). To detect differences in the expression of candidate genes between IAA treatment durations, one-way ANOVA was performed using IBM SPSS 20 software (IBM Corporation, USA). *P*<0.05 (*) and *P*<0.01 (**) were considered to indicate significant differences in expression.
